# Climatic Variables and Malaria Morbidity in Mutale Local Municipality, South Africa: A 19-Year Data Analysis

**DOI:** 10.3390/ijerph14111360

**Published:** 2017-11-08

**Authors:** Abiodun M. Adeola, Joel O. Botai, Hannes Rautenbach, Omolola M. Adisa, Katlego P. Ncongwane, Christina M. Botai, Temitope C. Adebayo-Ojo

**Affiliations:** 1 South African Weather Service, Private Bag X097, Pretoria 0001, South Africa; joel.botai@weathersa.co.za (J.O.B.); hannes.rautenbach@weathersa.co.za (H.R.); katlego.ncongwane@weathersa.co.za (K.P.N.); christina.botai@weathersa.co.za (C.M.B.); 2 Department of Geography, Geoinformatics & Meteorology, University of Pretoria, Private Bag X20, Hatfield 0028, South Africa; lolaadisa@yahoo.com; 3 School for Health Systems and Public Health, University of Pretoria, Pretoria 0002, South Africa; adebayotc@gmail.com

**Keywords:** malaria, climate, environment, morbidity

## Abstract

The north-eastern parts of South Africa, comprising the Limpopo Province, have recorded a sudden rise in the rate of malaria morbidity and mortality in the 2017 malaria season. The epidemiological profiles of malaria, as well as other vector-borne diseases, are strongly associated with climate and environmental conditions. A retrospective understanding of the relationship between climate and the occurrence of malaria may provide insight into the dynamics of the disease’s transmission and its persistence in the north-eastern region. In this paper, the association between climatic variables and the occurrence of malaria was studied in the Mutale local municipality in South Africa over a period of 19-year. Time series analysis was conducted on monthly climatic variables and monthly malaria cases in the Mutale municipality for the period of 1998–2017. Spearman correlation analysis was performed and the Seasonal Autoregressive Integrated Moving Average (SARIMA) model was developed. Microsoft Excel was used for data cleaning, and statistical software R was used to analyse the data and develop the model. Results show that both climatic variables’ and malaria cases’ time series exhibited seasonal patterns, showing a number of peaks and fluctuations. Spearman correlation analysis indicated that monthly total rainfall, mean minimum temperature, mean maximum temperature, mean average temperature, and mean relative humidity were significantly and positively correlated with monthly malaria cases in the study area. Regression analysis showed that monthly total rainfall and monthly mean minimum temperature (*R*^2^ = 0.65), at a two-month lagged effect, are the most significant climatic predictors of malaria transmission in Mutale local municipality. A SARIMA (2,1,2) (1,1,1) model fitted with only malaria cases has a prediction performance of about 51%, and the SARIMAX (2,1,2) (1,1,1) model with climatic variables as exogenous factors has a prediction performance of about 72% in malaria cases. The model gives a close comparison between the predicted and observed number of malaria cases, hence indicating that the model provides an acceptable fit to predict the number of malaria cases in the municipality. To sum up, the association between the climatic variables and malaria cases provides clues to better understand the dynamics of malaria transmission. The lagged effect detected in this study can help in adequate planning for malaria intervention.

## 1. Introduction

Malaria poses the biggest threat with about 40% of the world’s population at risk of infection among other vector-borne diseases [1]. Malaria is responsible for about 300–500 million cases and about one million deaths annually, with a larger percentage of the cases and deaths occurring in sub-Saharan Africa [2]. According to [3], about 4.9 million of the South African population representing 10% of the total population live in the malaria-endemic area. Malaria is majorly endemic in three provinces, namely Limpopo, Mpumalanga, and KwaZulu-Natal, although occasionally few major occurrences are sighted in the Northern Cape and North-West provinces along the Orange and Molopo Rivers as a result of the provision of suitable breeding habitats for mosquitoes to survive (Department of Health, South Africa, 2007). *Plasmodium falciparum* accounts for about 95% of the total malaria infections in South Africa through *Anopheles arabiensis* as the major local vector.

Potential risk factors for malaria transmission such as population movement, population immunity, availability of suitable vector habitats, malaria control measures, social and economic status (a reflection of housing types and housing conditions), environmental factors (land use/cover; vegetation, water body, irrigation/farmlands and elevation) and climatic factors (rainfall, temperature, relative humidity) have been shown to have a significant impact on the transmission of the disease [4,5,6]. According to [7], the severity of health risks such as malaria, associated with climate and its changes, will depend on the ability of public health and safety systems to address or prepare for these risks, as well as factors such as an individual's behavior, age, gender, and economic status. Impacts will vary based on where a person lives, how sensitive they are to health risks, how much they are exposed to climate impacts, and how well they and their community are able to adapt to these impacts.

With the recent surges in malaria morbidity, there is no better time to address the urgent need for the development of operational malaria early warning systems to predict when, where, and what magnitude of malaria epidemics might occur, with sufficient lead-time to target scarce resources for effective control measure. More often than not, meteorological conditions, such as high rainfall and/or high temperature, are cited as the causing factors for malaria epidemics [8,9,10]. Many efforts have been made to predict malaria epidemics by using climatic variables on a local and/or global scale [5,6,11,12]. However, several studies have reported variations in the relationship between climatic factors and malaria occurrences from one geographic space to another. This suggests that one or more climate factors are more important than others for malaria prediction. This has led to subtle agreement about the relative importance and predictive value of different climatic factors. For instance, temperature was reported as a strong predictor of transmission of the malaria parasite *Plasmodium falciparum* over the entire continent of Africa [12]. On the other hand, rainfall has been reported to be the major climatic factor driving malaria incidence in India [5], Mozambique [6], Ethiopia [10], and in Niger Sahel [13].

Studies in other malaria endemic regions of South Africa (i.e., Kwazulu-Natal and Mpumalanga Provinces), have shown that the incidence of malaria is related to local climatic variables. In this regard, temperature and rainfall are the most common climate variables correlated against malaria cases [14,15,16,17,18]. KwaZulu-Natal and Mpumalanga Provinces have received relatively more research attention compared to the neglected Limpopo province, particularly the rural remote north-eastern area, which continually records a high number of malaria cases.

**Figure 1:**
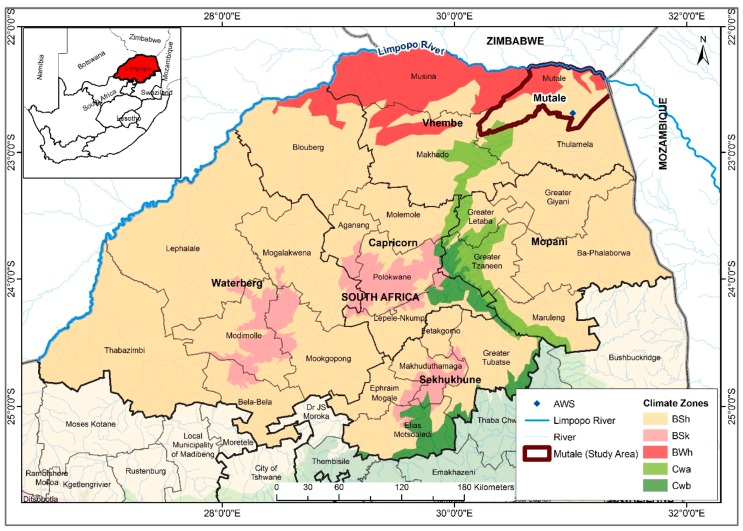
Map of Limpopo Province, South Africa, depicting the climatic zones and the Limpopo River (blue line) and its distributaries, including Mutale local municipality in brown boundary and the location of the Automatic Weather Station (AWS), Hot semi-arid climate (BSh), Cold semi-arid climate (BSk), Hot desert climate (BWh), Humid subtropical climate (Cwa) and Subtropical highland oceanic climate (Cwb).

Previous work by [19] showed that the incidence rates in Limpopo was related to both temperature and rainfall and hence laid the foundation for relating climatic variables with malaria cases in the province. Therefore, in view of developing an operational malaria early warning system, it is imperative to critically examine the association and lag structure between climatic variables and malaria incidence in the Mutale-Vhembe region of Limpopo Province (Figure 1). Therefore, the present study is designed with the objective of determining the role of each of the local climatic variables (monthly: total rainfall (Rain), mean maximum temperature (Tmax), mean minimum temperature (Tmin), and mean temperature (Tavg), as well as mean relative humidity (RH)) on malaria occurrence in the Mutale-Vhembe region of the South African Limpopo Province in order to model the effects of the variables on malaria occurrence and predict future cases of malaria in the Mutale local municipality.

## 2. Materials and Methods

### 2.1. Study Area and Population

Mutale local municipality is located in the far north-eastern part of Limpopo Province in South Africa, bordering Zimbabwe and Mozambique. It lies between latitudes 22°3′ S and 22°9′ S and longitudes 30°2′ E and 31°4′ E. Mutale is one of the four local municipalities in Vhembe district municipality of Limpopo Province. The population of Mutale is estimated to be 91,870 [3] within an area of 3886.14 Km^2^ at a mean altitude of 850 m. The area is drained by the Limpopo River and its tributaries. According to Koppen Geiger climate classification, about 78% of the area is classified as hot semi-arid climate (BSh), 7% cold semi-arid climate (BSk), 8% as hot desert climate (BWh), 4% as humid subtropical climate (Cwa) and 3% as subtropical highland oceanic climate (Cwb) [20]. The average annual temperature is 24.6 °C, the minimum average temperature is 18.9 °C in June, and the maximum average temperature is 28.2 °C in January. About 420 mm of precipitation is received annually. The least amount of rainfall is received in July, with an average of 2 mm; while most of the precipitation falls in January. The average annual RH is about 77.4%. Figure 1 illustrates the geographical location of Mutale local municipality, its climate classification, and the position of the Automatic Weather Station (AWS) from which climate data used for analysis was collected in Limpopo Province of South Africa.

### 2.2. Datasets

Both malaria and climate data used in this study spans a period of 19 years (from 1998 to 2017). Daily malaria data were collected by the Limpopo provincial malaria control programme and stored in their malaria information system in Tzaneen. The data included both active and passive surveillance malaria case patients, diagnosis date, sex, age, district and local council where the patient resides, source country or province in South Africa where the patient presumably contracted malaria, and malaria deaths reported. Daily climate variables such as daily mean temperature, minimum temperature, maximum temperature (°C), relative humidity (%), and rainfall (mm) were collected from a South African Weather Service (SAWS) AWS located within the study area. The malaria and climate data were aggregated to monthly data since aggregation to monthly data help in minimizing errors due to data gap filling.

### 2.3. Ethical Statement

Malaria data analyzed in the study were mined from the Limpopo provincial malaria information system and no further information was sought from the patients. The climate data was approved by the climate services unit of SAWS.

### 2.4. Data Analysis

All statistical analyses were performed with Microsoft Excel and R [21]. The climate data were checked for missing values. Missing values were replaced by the average of nearest values using the Fill and trend functions of Excel. The monthly mean of climatic variables Tmax, Tmin, Tavg, RH, and monthly total amount of rainfall were treated as independent variables, and monthly malaria case was treated as a dependent variable. Descriptive statistics using boxplot was performed to explore the climatic variables and malaria case. Spearman’s correlation analysis was performed to determine the relationship between the malaria case and monthly climatic variables, and for the identification of climatic variables that mostly influence or are the dominant predictor of malaria incidence. Time series analysis was then conducted on the data set using the Box-Jenkins approach [22]. As building blocks of a time series, the seasonality, trend, and cycle component of the series were evaluated by decomposing the data. The malaria case data was logarithmically transformed and differenced once to induce constant variance and stationarity. Hence, *Y’* = (*Y*_1_, *Y*_2_, …, *Y_n_*) is the vector of the natural logarithms of the monthly number of cases of malaria cases from 1998 to 2017, in which the value 1 was added to deal with the logarithm of zero values in cases of non-occurrence of malaria in a given month. Augmented Dickey-Fuller (ADF) test, a formal statistical test for stationarity [23], was used to check for stationarity. The ADF tests if the change in *Y* can be explained by lagged value and a linear trend. A stationary time series is a time series without trend, having a constant mean and variance over time, a condition to be satisfied in using the Box-Jenkins approach.

The transformed time series of malaria cases were analysed to identify the auto-regressive, moving average and differencing orders of the Seasonal Autoregressive Integrated Moving Average (SARIMA) model. The autoarima function, which searches through combinations of order parameters and selects the order that best fits the model and manual selection of model, was performed. The Autocorrelation Function (ACF) and the Partial Autocorrelations Function (PACF) were assessed. The Akaike Information Criterion (AIC) values (see for example [24]) were used to determine the goodness-of-fit of the models. Since lower AIC values indicate better fit, the model with the lowest AIC was selected. The model adequacy was proven by plots of the histogram and ACF of the standardized residuals and the Ljung-Box Q statistic [25]. The ACF of the residuals and Ljung-Box Q statistics were used to test the randomness of the residuals. The time series data were divided into training (80%) and testing (20%) data in order to allow for cross-validation. Cross-validation is primarily a way of assessing the predictive performance of a model against a set of data not used in estimation. The testing data left out of model estimation processes was used to make out-of-sample prediction and forecast for 12 months. The performance measures of prediction accuracy were done using the Mean Absolute Percentage Error (MAPE), the Root Mean Squared Error, and the adjusted R^2^.

The SARIMAX model was used to forecast the monthly time series of malaria case using Box-Jenkins SARIMA approach and the Multiple Linear Regression (MLR). The SARIMAX model is essentially a linear regression model that uses SARIMA model type process with exogenous variables, called SARIMAX (p,d,q) (P,D,Q)_S_ (X), where X is the vector of external variables. A multi linear regression equation used to model the external variables is expressed as

(1) $$Y_{t}=\beta_{0}+\beta_{1}X_{1,t}+\beta_{2}X_{2,t}+\ldots +\beta_{k}X_{k,t}+\omega_{t}$$

where, *X*_1,*t*_, *X*_2,*t*_, …, *X_k,t_* = the *k*th exogenous input variable at time *t* corresponding to the dependent variable *Y_t_*; *β*_0_, *β*_1_, …, *β_k_* = regression coefficient value for the *k*th exogenous (explanatory) input variable; *ω_t_* = a stochastic residual, i.e., the residual series that is independent of input series [26].

The SARIMA model is particularly useful in situations where stationary and non-stationary time series data exhibit seasonality periodic fluctuations. The use of SARIMA to create time series forecast yields a reliable output when there are no data outliers. Outliers are known to have a potential impact on the estimates of the model parameters. Outliers in a time series are often indicative of significant events or exceptions, hence providing useful information to the process. Consequently, it is imperative to examine external variables, which provide meaningful answers to the outlying data. The SARIMAX model is useful in cases were residuals may be suspected to exhibit a seasonal trend or pattern. With the substitution of residual series in the multi linear regression Equation (1), the general SARIMAX model equation is expressed as

(2) $$Y_{t}=\beta_{0}+\beta_{1}X_{1,t}+\beta_{2}X_{2,t}+\ldots +\beta_{k}X_{k,t}+\left(\frac{\theta_{q}(B)\Theta_{Q}(B^{S})}{\phi_{p}(B)\Phi_{P}(B^{S}){\left(1-B\right)}^{d}{\left(1-B^{S}\right)}^{D}}\varepsilon_{t}\right)$$

## 3. Results

**Figure 2:**
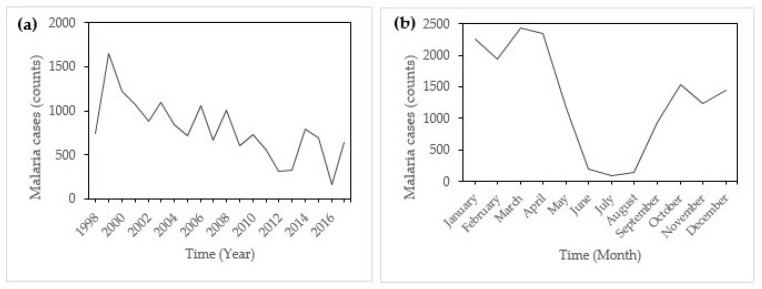
(**a**) Annual and (**b**) monthly cases of malaria in Mutale local municipality, Limpopo, January 1998–May 2017.

During the period under investigation, January 1998 to May 2017, a total of 15,739 malaria cases were recorded in Mutale local municipality. This number of cases accounts for 27.1% of the total malaria cases in the Vhembe district municipality (57,974 malaria cases). Within this period, a total of 11,989 (76.2%) malaria cases were locally transmitted, 538 (3.4%) were imported malaria, 1995 (12.7%) were source not indicated, 1057 (6.7%) were untraceable, and cases with an uncompleted investigation totalled 160 (1%). 14,462 (91.9%) of the cases were detected by passive surveillance and 1275 (8.1%) by active surveillance; 2 cases had no indication for surveillance type. Gender wise, the male gender account for 8566 (54.4%) and female 7170 (45.6%) of the cases. Three cases had no indication of gender type. Figure 2a shows the annual incidence of malaria in Mutale local municipality from January 1998 to May 2017.

There was a remarkable peak in incidence in 2000 with a remarkable decline afterward. Figure 2b indicates that there was monthly variation in the incidence of malaria in Mutale, with incidence rising from September and droping after May. Peak malaria incidence is noticeable in January and little incidences are recorded in June, July, and August (JJA), which are austral winter months.

**Figure 3:**
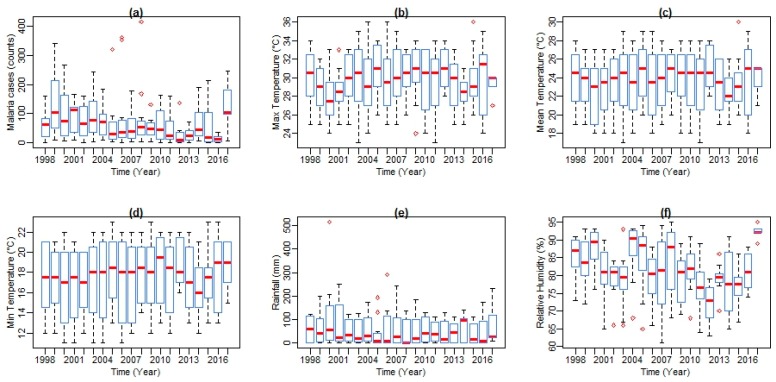
Boxplots of annual malaria cases (counts) (**a**), maximum (**b**), mean (**c**), minimum (**d**), temperature and rainfall (**e**), and relative humidity (**f**). Values indicate minimum, first quartile, median, third quartile, and maximum from bottom to top.

**Figure 4:**
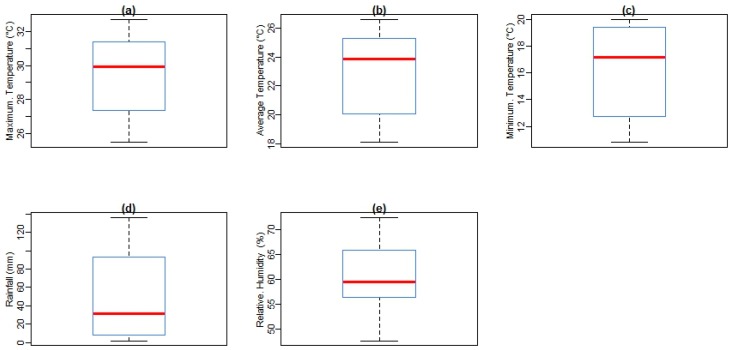
Boxplot of average monthly climatic variables (**a**) maximum, (**b**) mean, (**c**) minimum temperatures, (**d**) rainfall, and (**e**) relative humidity in Mutale local municipality, Limpopo, 1998–2016.

Figure 3 shows box plots of malaria cases and climate variables with the quantile values for minimum, first quartile, median, third quartile, and maximum in Mutale for the period under investigation. At a monthly Tavg of 26.6 °C, December is the hottest month of the year. At 18.1 °C on average, July is the coldest month of the year. Between the driest and wettest months, the difference in rainfall is 110 mm. The variation in annual temperature is around 8.2 °C. In addition, over the period under investigation, as shown in Figure 4, the monthly mean average of Tmax was 29.5 °C (Standard Deviation (SD) = 3.15), the monthly mean average of Tmin was 16.2 °C (SD = 3.49), monthly average Tavg was 22.8 °C (SD = 2.92), monthly average rainfall was 49.8 mm (SD = 5.45), and monthly mean average of RH was 61.7% (SD = 8.86). In addition, over the period considered in the study area, the highest mean Tmax received is 36.0 °C, highest mean Tmin is 23.0 °C, highest mean Tavg is 30.0 °C, highest mean RH is 95%, and the highest total rainfall received is 515 mm, recorded in February 2000.

**Figure 5:**
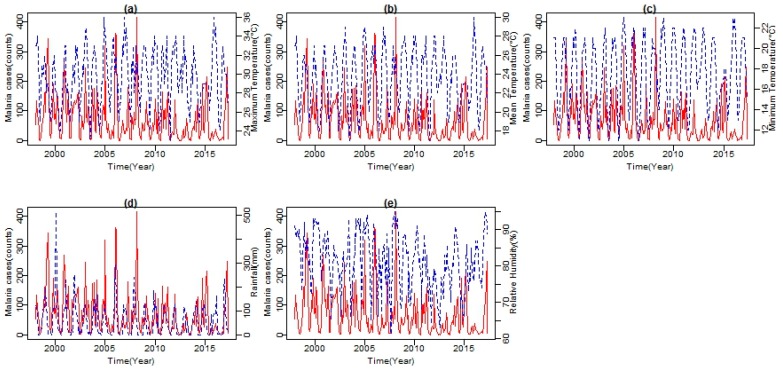
The plot of time series of malaria counts indicated on the left of the Y-axis (red line) and time series of (**a**) maximum, (**b**) mean, (**c**) minimum temperatures, (**d**) rainfall and (**e**) relative humidity on the right of the Y-axis (blue line).

As shown in Figure 5, both climatic variables’ time series and malaria cases series from 1998 to 2017 exhibited seasonal patterns. Climatic variables are shown in blue lines and malaria cases in red line. All series showed a number of peaks and fluctuations. The peaks in the series seem to be separated by more than a few months, indicating a seasonal pattern in the variable. From the series, an increasing trend in temperature, with more observations of higher than normal temperatures since 2003, is noticeable. A similar trend is exhibited by both Tmax and Tmin. However, Tmax exhibits a much steeper trend with a slight increase in Tmax observed between November and February (see Figure 4). The mean RH lies well above 61%. High and low values of RH are recorded during the summer months with the peak in February and in the winter months with the lowest in August, respectively. Total rainfall amount also followed a strongly seasonal pattern during the period under investigation. Between 1998 and 2017, the months of November, December, and January recorded the highest amount of rainfall during the year.

**Figure 6:**
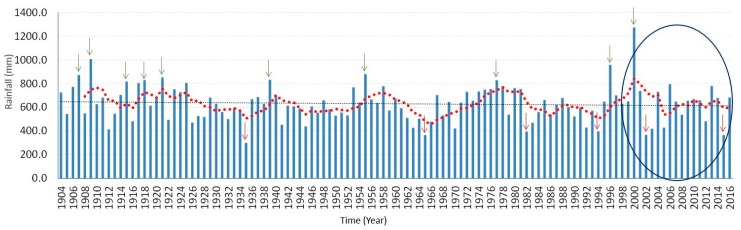
Long time-series of total annual rainfall (mm) over the entire Limpopo Province for 113 years (1904–2016). The blue line is the annual total rainfall: January–December (mm); the red, dashed line is the 5-year running mean (mm); the black, horizontal line is the linear trend line; the green arrow is the years with rainfall above 800 mm, while red arrow is years with rainfall below 400 mm.; and the blue oval is the period of year under investigation.

The cumulative rainfall amount per month in these months is above 460 mm. Furthermore, the analysis revealed a strong association between years of high malaria cases and years of above normal rainfall, which significantly increased RH to an average of about 65% and both Tmin and Tmax values at an average of 18 °C and 26 °C, respectively. For the period under investigation, positive correlations are found in two to three months after the tropical storm must have occurred. Four major periods of high malaria incidences found are the 1999–2000, 2007–2008, 2010–2011, and the recent 2016–2017 with an average of (*R* = 0.79; *p* < 0.001) (see Figure 5d). Shown in Figure 6 is the time series of total annual rainfall over the entire Limpopo Province from 1904 to 2016. Previous study has shown that the years of above normal rainfall are associated with positive sea surface temperature anomalies, La Niña conditions in the Pacific Ocean as well as tropical cyclones from the Mozambique Channel [27]. In summary, at an Tavg of 26.6 °C, December is the hottest month of the year. At 18.1 °C on average, July is the coldest month of the year. Between the driest and wettest months, the difference in precipitation is 110 mm. The variation in annual temperature is around 8.2 °C.

**Figure 7:**
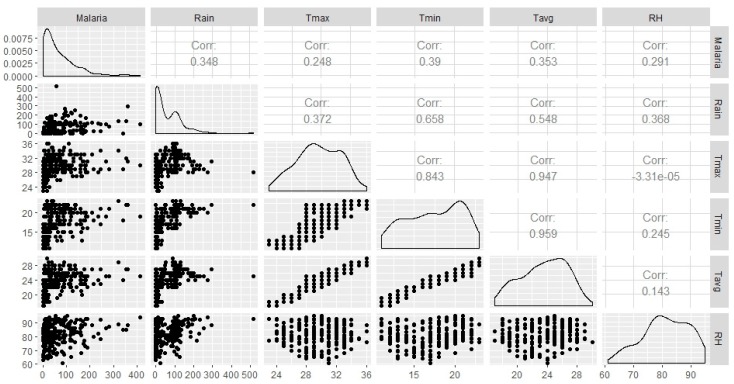
Spearman’s correlation analysis between climatic variables; rainfall (mm) Rain; maximum temperature (°C) Tmax; minimum temperature (°C) Tmin; average temperature (°C) Tavg; relative humidity (%) RH; and malaria counts.

Spearman’s correlation analysis indicates that statistically significant association between all climatic variables (Tmax, Tmin, Tavg, Rain and RH) and monthly malaria cases (Figure 7). Tmin shows the highest correlation (*R* = 0.39; *p* < 0.001), followed by Rain and Tavg (r = 0.35; *p* < 0.001, r = 0.35; *p* < 0.001), followed by RH and Tmax with (*R* = 0.29; *p* < 0.001, r = 0.25; *p* < 0.001), respectively. The relationship between monthly malaria cases and the climatic variables examined at zero to three months lagged-periods show different effects. At zero-month lag time, climatic variables did not show a strong relationship with malaria cases. However, strong, statistically significant correlations were seen between the climatic variables and monthly malaria cases when the climatic variables time-series lagged malaria time series by at least one month (Table 1). Rainfall is negatively and significantly associated with malaria cases at lags 0 and 1-month. Most significant associations were observed at two-month lag time. Rainfall shows the highest association at two-month lag time (*R* = 0.49; *p* < 0.001), followed by Tmin (*R* = 0.43; *p* < 0.001), Tavg (*R* = 0.42; *p* < 0.001), and RH (*R* = 0.39; *p* < 0.001). Different combinations of climatic variables with malaria cases were performed. Total monthly rainfall and monthly mean minimum temperature, with a two-month lagged effect, were found to be the most significant climatic variables for malaria transmission in Mutale municipality (*R* = 0.55; *p* < 0.001).

**Figure 8:**
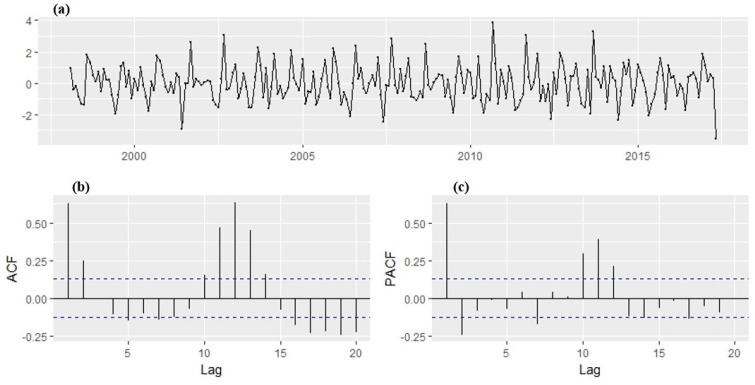
(**a**) Time series of logarithmic and differenced malaria cases between January 1998 and May 2017, (**b**) the Autocorrelation function (ACF), and (**c**) Partial Autocorrelation function (PACF) were used to identify the appropriate order of Autoregressive (AR) and Moving Average (MA).

With the transformation, a stabilized variance is achieved with no trend overtly observed in the series. The ADF test result indicated that the transformed time series of monthly malaria cases is stationary, i.e., the p-value is less than 0.05 meaning that there is no unit root. Hence, the ADF test rejects the null hypotheses of non-stationarity. The plot of the transformed series (Figure 8) shows no visible strong trend, suggesting that differencing order of 0 term is sufficient for the model. The 95% significance boundaries are plotted as dotted blue lines. Significant spikes in the auto and partial correlation at lag 1, 2, 11, and 12 can be noticed. The spikes at lag 12 might suggest the presence of a seasonal pattern, perhaps as months of the year. The plots suggest SARIMA (2,1,2) (1,1,1)_12_ confirmed with the autoarima function as the best forecasting model that fit the training dataset well. The AIC values for the SARIMA models fitted to the malaria cases are shown in Table 2. From the coefficients obtained, the return equation can be written as:

(3) $$Y_{t}=0.5283*Y_{\left(t-1\right)}-0.0373*Y_{\left(t-2\right)}-0.9898\varepsilon_{\left(t-1\right)}+0.5704\varepsilon_{\left(t-2\right)}$$

**Figure 9:**
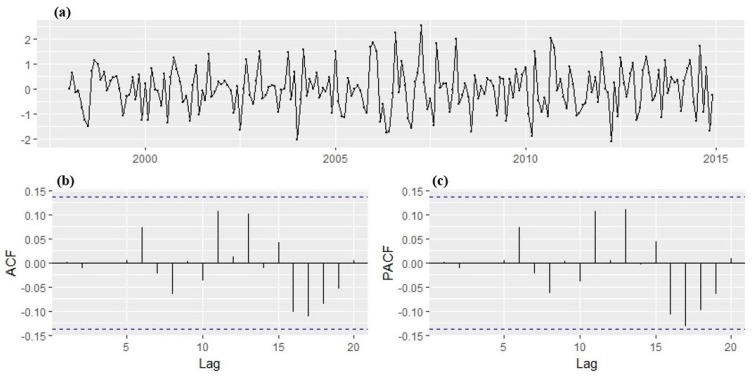
(**a**) Residual of the time series, (**b**), the Autocorrelation function (ACF), (**c**) Partial Autocorrelation function (PACF) function of log and differenced time series of malaria cases in Mutale local municipality (January 1998–December 2014).

The diagnostic plots of the fitted model are given in Figure 9. With all lags of the ACF and PACF within the 95% significance boundaries, the residuals, therefore, suggest that the correlations are close to zero and are normally distributed centered at 0. This implies that the residuals do not deviate significantly from the zero mean white noise process. Hence, this indicates that the model passed the Ljung Box test approach test, model fits the data well, and it is therefore suitable to make a forecast.

**Figure 10:**
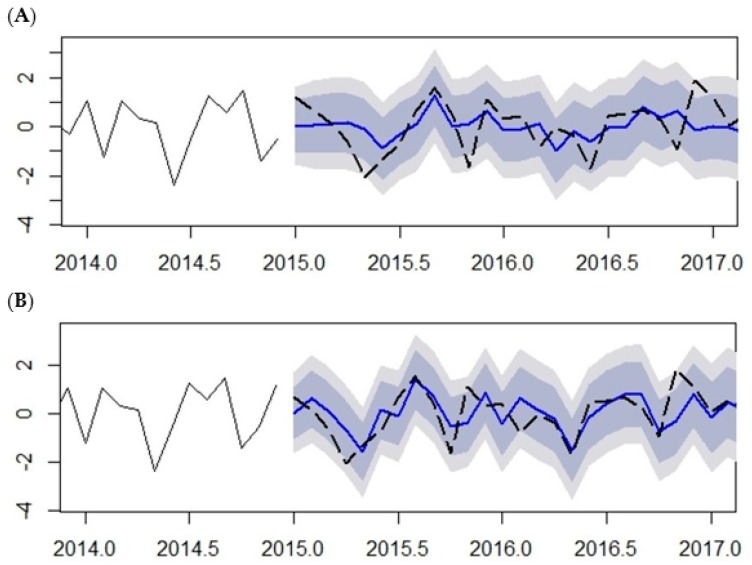
Actual (observed-dashed black line) and predicted (fit-blue line) transformed values of malaria cases in Mutale municipality from January 2015 to May 2017. (**A**) Without climatic variables and (**B**) with climatic variables as exogenous variables.

Out-of-sample prediction malaria cases from January 2015 to May 2017 using the SARIMA (2,1,2) (1,1,1)_12_ model was performed and compared with the 20% observation testing data left out of the modelling procedure (January 2015 to May 2017) (See Figure 10 and Table 3). The plots illustrate the predicted malaria cases fitted with observed malaria cases without climatic variables (Figure 10A) and with climatic variables (Figure 10B) as exogenous variables. The predictions made with climatic variables, either over-prediction (predictions greater than observed) or under-prediction (predictions less than observed), are within 10% of the observed malaria cases. The dashed black line is the observed cases while the blue line is the predicted cases. The dark gray shaded area is the 80% prediction interval and the light gray shaded area is the 95% prediction interval.

The comparison between the predicted and observed number of malaria cases is shown in Table 3. The predicted values are relatively close to the observed values; this result indicates that the model provides an acceptable fit to predict the number of malaria cases. The prediction shows continued high amount of malaria cases further down till July, which is normally a low malaria-transmission period, indicating a shift in the malaria season. The performance measures of the prediction accuracy are shown in Table 4.

## 4. Discussion

Malaria transmission is influenced by many factors, which include the abundance of malaria vector, the survival rate and longevity of the mosquitoes, the parasite’s development rate in the mosquitoes, the mosquitoes biting rate, and human susceptibility to parasites governed by human behaviour and immunity. Other factors include economic and social factors such as population movement, housing conditions, sanitation condition, and malaria control measures. However, because of its dependence on the environment and climate, its variability is considered as a major determinant of malaria transmission. This is because of climate impacts on the incubation rate of the *Plasmodium* and the breeding activity of *Anopheles*.

Increase in temperature is expected to increase the prevalence as well as transmission of malaria because it shortens the interval between mosquitoes’ blood meals, thus reducing the incubation period of the parasite in mosquitoes leading to a reduced time for the production of new mosquito generations. Temperatures range between 20 °C and 30 °C, shortening the extrinsic incubation period of the parasite. At 16 °C, larval development may take more than 45 days leading to a reduction in the number of mosquitoes [12]. Hence, temperatures below 16 °C and above 30 °C negatively impact on the survival of the mosquitoes. On the other hand, rainfall influences the aquatic stage of the mosquitoes’ life cycle. The laying of mosquito eggs, the development to larvae, and the development into adults require aquatic breeding sites created through rainfall. Rainfall, in addition, increases relative humidity to sustain the longevity of the adult mosquito. Consequently, temperatures between 20 °C and 30 °C, RH above 65%, and adequate amount of rainfall (cumulative average of about 400 mm) are optimal for the survival of *Anopheles* to acquire and transmit the parasite. Hence, with a monthly mean of Tmin as 17.45 °C, monthly highest of Tmin as 23 °C, and monthly lowest of Tmin as 11 °C recorded over the period of study, it can be implied that the temperature ranges create an ideal location for malaria vector breeding.

Positive correlations between monthly malaria cases and all climatic variables were found in Mutale local municipality. Mean Tmin has the highest correlation coefficient with malaria cases. This indicates that Tmin influences the transmission of malaria more than any other climatic variables in the Mutale local municipality. The combination of total monthly rainfall and monthly mean minimum temperature, with a two-month lagged effect, were found to be the most significant climatic variables for predicting malaria transmission in Mutale municipality with an *R*^2^ = 0.65 compared to *R*^2^ = 0.54 (mean maximum temperature and rainfall), *R*^2^ = 0.51 (mean average temperature and rainfall), and *R*^2^ = 0.49 (mean relative humidity and rainfall). This result is similar to what was reported by [15], in the study in Mpumalanga Province in South Africa.

The negative but statistically significant association between rainfall and malaria cases at lag 0 and 1-month may be due to breeding sites being washed off by high rainfall (flooding) [28]. The significant relationships noticed at time lag above 1-month between the occurrence of malaria and the associated climatic variables, such as rainfall and minimum temperature at two months, would suggest that climatic conditions in a given year (onset of early rainfall particularly those associated with La Niña conditions and tropical cyclones) may affect malaria transmission in the following year. The periods of high rainfall coupled with low temperature and high relative humidity favour saturated soil moisture content leading to longer life span for pockets of water, which could lead to the persistence of larval habitats [29,30]. Although La Niña years and tropical cyclones which are associated with flooding could wash away mosquito breeding sites, pockets of standing water created after the flood can generate new breeding sites. This implies that ideal climatic and environmental conditions could result in increased vector population with potential for malaria transmission within few weeks. However, habitat preference of the local vector species is a strong determinant [31]. The result of the two-month lagged effect is realistic in view of the malaria cycle consisting of three components: (i) the growth of the *Anopheles* female mosquito from egg to adult to parasite transmission; (ii) the development of the *Plasmodium* parasites (gametocyte to sporozoites) that are able to infect humans; and (iii) the incubation period in the human host from infection to malaria symptoms [32]. Hence, malaria occurrence can be expected to be at a peak at about one and a half and two months after the onset of rain. Similar results were found in Mozambique [6,33], Zimbabwe [34], and Ethiopia [11].

The SARIMA (2,1,2) (1,1,1)_12_ model was found to be the best fit model for a reliable forecast. Two models were developed for this study; the output of models is shown in Figure 9. The model with the inclusion of all climatic variables shows a better prediction performance of accuracy of 72%, while the model without climatic model has an accuracy of 51%. This implies that 71% of the variance in malaria cases can be explained by variance in the climatic variables. The result is comparable with results in India [5], Mozambique [6], and Burundi [35]. However, this result is far lower than what was reported in world malaria report, where 90% of the variance in malaria cases can be explained by environmental factors. Other contributing factors may include economic and social factors such as population movement, housing conditions, sanitation condition, and malaria control measures and environmental factors such as land use type and topography, which are not considered in this present study.

## 5. Conclusions

Although malaria transmission shows seasonality in accordance with climate in South Africa, only a few studies have been conducted in assessing the relationship between malaria cases and climatic variables at a local level, particularly in the north-eastern part of South Africa. Therefore, the aim of this study was to find the relationship between historical malaria cases and climatic variables and hence develop the SARIMA model to predict the expected number of malaria cases per month based on observed malaria cases and climatic variables as the predictors. The study found that total monthly rainfall and monthly mean minimum temperature, with a two-month lagged effect, are the most significant climatic variables for malaria transmission in the Mutale municipality (r = 0.55; *p* < 0.001). In addition, this study found a relationship between years of high malaria cases and La Niña events, as well as tropical cyclones from the Mozambique Channel. A detailed analysis for comprehensive reporting of this association is in view by the same authors.

The lag-time effect between malaria occurrence and climatic events is essential for the prediction of malaria cases and can be used for planning and implementing malaria control and interventions [36]. The model can be improved by incorporating interventions such as insecticide residual spraying, land use/cover characteristics, migration pattern, housing conditions, and biology of the vector in preparation for the development of an integrated malaria early warning.

Therefore, in view of the above and the national department of health’s target of 2020 as the malaria elimination year, an integrated multidisciplinary and coordinated response is required to identify all epidemiological and ecological factors driving the persistence of malaria in the northern region of South Africa/the southern region of Zimbabwe.

## Figures and Tables

**Table 1:** Correlation between malaria cases and climatic variables at lags of 0, 1, 2, and 3 months.

Climatic Variables	Lag Time	*R*	*p*-Value
**Rainfall**	0 month	−0.29	<0.001
	1 month	−0.27	<0.001
	2 months	0.53	<0.001
	3 months	0.49	<0.001
**Tmax**	0 month	0.24	0.109
	1 month	0.42	<0.001
	2 months	0.41	<0.001
	3 months	−0.34	>0.012
**Tmin**	0 month	0.25	0.058
	1 month	0.41	<0.001
	2 months	0.57	<0.001
	3 months	0.54	<0.001
**Tavg**	0 month	0.52	<0.001
	1 month	0.45	<0.001
	2 months	0.42	<0.004
	3 months	0.09	>0.191
**RH**	0 month	0.28	<0.001
	1 month	0.37	<0.001
	2 months	0.39	<0.001
	3 months	0.10	>0.011

**Table 2:** Akaike information criterion values considering different Seasonal Autoregressive Integrated Moving Average (SARIMA) models.

Model Type	AIC Values
(2,1,2) (1,1,1)_12_	516.65
(1,1,0) (2,1,1)_12_	560.24
(1,1,1) (0,1,1)_12_	605.36
(2,1,0) (2,1,0)_12_	546.17
(2,1,1) (2,1,0)_12_	517.83
(2,1,0) (2,1,0)_12_	547.63

**Table 3:** The observed number of malaria cases in January 2015–May 2017 in Mutale local municipality, and the out-of-sample predicted values obtained from SARIMA (2,1,2) (1,1,1)_12_ model with climatic variables as exogenous variables.

Year	Malaria Cases	Months												
		Jan.	Feb.	Mar.	Apr.	May.	Jun.	Jul.	Aug.	Sept.	Oct.	Nov.	Dec.	Total
**2015**	**Observed**	91	181	215	117	14	3	1	3	18	30	5	17	695
	**Predicted**	79	189	224	126	21	2	1	2	29	21	3	11	708
**2016**	**Observed**	24	37	16	15	10	1	2	4	9	12	4	31	165
	**Predicted**	16	48	17	14	6	1	2	3	4	5	3	29	148
**2017**	**Observed**	98	105	182	247	6	-	-	-	-	-	-	-	638
	**Predicted**	95	102	144	198	29	31	20	22	18	7	6	31	703

**Table 4:** Performance measures of the model for out-sample prediction.

Model Type	Predicting without Climatic Variables			Predicting with Climatic Variables		
SARIMA (2,1,2) (1,1,1)_12_	MAPE	RMSE	Adjusted R^2^	MAPE	RMSE	Adjusted R^2^
	26.674	18.562	0.512	22.430	15.684	0.715

Mean Absolute Percentage Error (MAPE), Root Mean Squared Error (RMSE).
